# Supplementary material for: Effects of Parental Migration on Dental Caries of Six- to Eight-Year-Old Children Using Structural Equation Modeling
Source: Int J Environ Res Public Health. 2022 Oct 18;19(20):13470. doi: 10.3390/ijerph192013470 (PMC9602841; doi:10.3390/ijerph192013470)
Supplement: Supplementary file 1 [file ijerph-19-13470-s001.zip › ijerph-1931705-supplementary.pdf]

Effects of Parental Migration on Dental Caries of 6- to 8-years-old Children Using Structural Equation Modeling

Sichen Liu [1], Virasakdi Chongsuvivatwong [2], Shinan Zhang [3] and Angkana Thearmontree [1,*]

[1] Improvement of Oral Health Care Research Unit, Community Dentistry Division, Department of Preventive Dentistry, Faculty of Dentistry, Prince of Songkla University, Songkhla 90110, Thailand
[2] Department of Epidemiology, Faculty of Medicine, Prince of Songkla University, Songkhla 90110, Thailand
[3] Department of Dental Public Health, School of Stomatology, Kunming Medical University, Kunming 650032, China
* Correspondence: angkana.dent@gmail.com

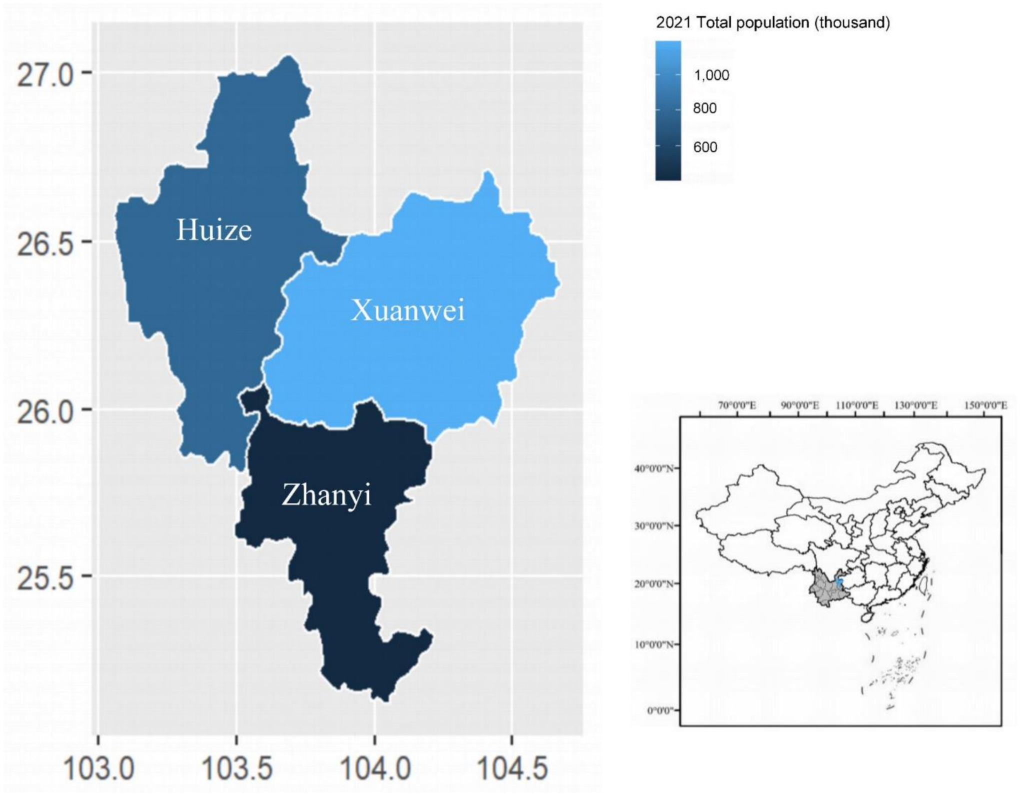

Figure S1. Map of the research areas: rural areas of the Yunnan province in China

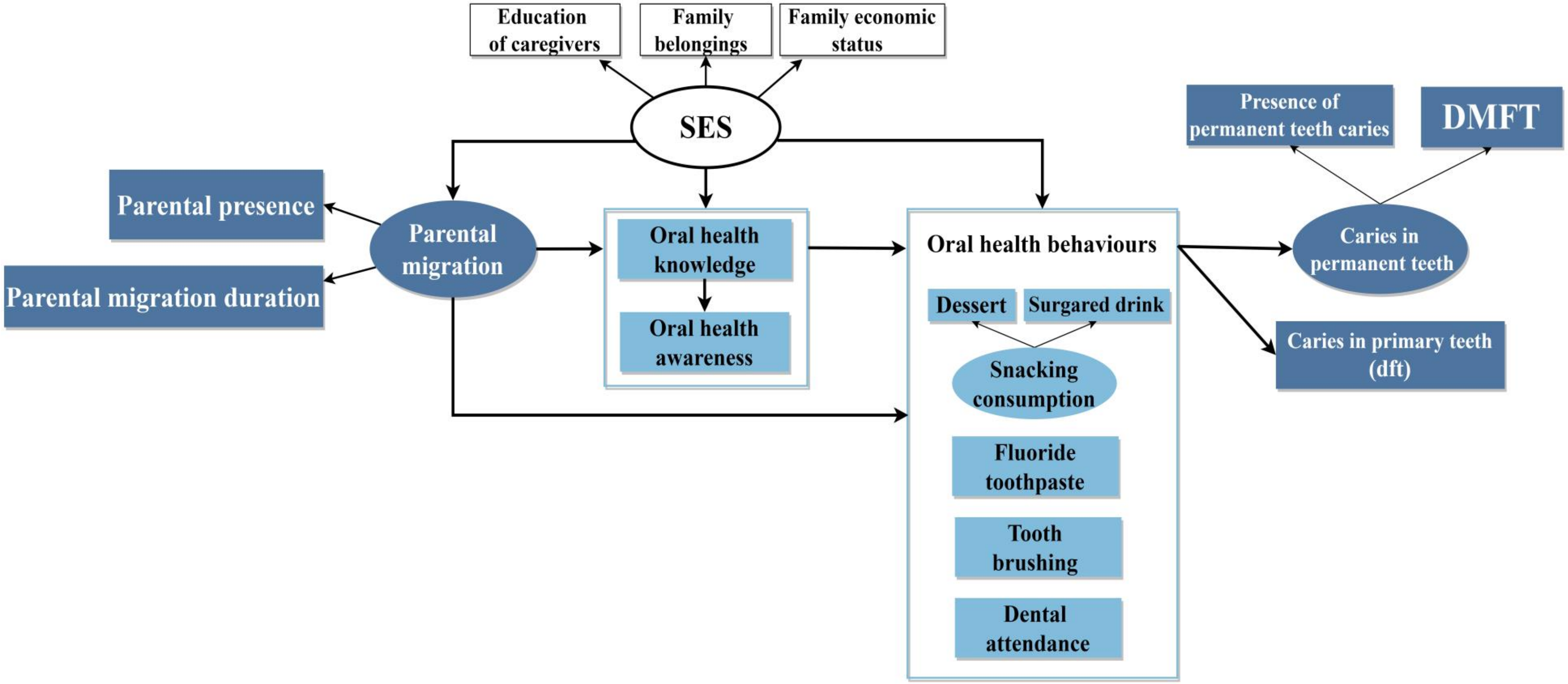

Figure S2. Path analysis model between parental migration and dental caries

Table S1. Variables in the path analysis model

Variables	Items	Code	Status
**Latent variables**			
Parental migration	Parental migration duration	(1)	No;
		(2)	Less than six months;
		(3)	Six to twelve months;
		(4)	More than twelve months.
	Parental presence	(1)	Both parents;
		(2)	Only mothers;
		(3)	Only fathers;
		(4)	Non-parents.
SES	Family economic status	(1)	Income less than expense;
		(2)	Income equal expense;
		(3)	Income more than expense.
	Family belongings		Count (0-9 items).
	Education of caregivers	(1)	No formal schooling;
		(2)	Primary school completed;
		(3)	Secondary school completed;
		(4)	High school completed;
		(5)	Vocational school;
		(6)	College / university completed.
Snacking consumption	Sugared drink	(1)	Few / Never / Don’t know;
		(2)	Once a day;
		(3)	Twice or more a day.
	Dessert	(1)	Few / Never / Don’t know;
		(2)	Once a day;
		(3)	Twice or more a day.
Caries in permanent teeth	Presence of permanent teeth caries	(0)	No;
		(1)	Yes;
	Number of permanent teeth caries (DMFT)		Count.
Caries in primary teeth	Number of primary teeth caries (dft)		Count.
**Observed variables**			
Brushing frequency		(1)	Never brush;
		(2)	Not brush every day;
		(3)	Once;
		(4)	Twice or more.
Fluoride toothpaste use		(0)	No;
		(1)	Yes.
Dental attendance		(1)	Never;
		(2)	Within 6 months;
		(3)	6 to 12 months;
		(4)	More than 12 months.
Oral health awareness of caregivers		(0)	Not appropriate (Total score: 0-2);
		(1)	Appropriate (Total score: 3-4).
Oral health knowledge of caregivers		(0)	Not appropriate (Total score: 0-4);
		(1)	Appropriate (Total score: 5-7).

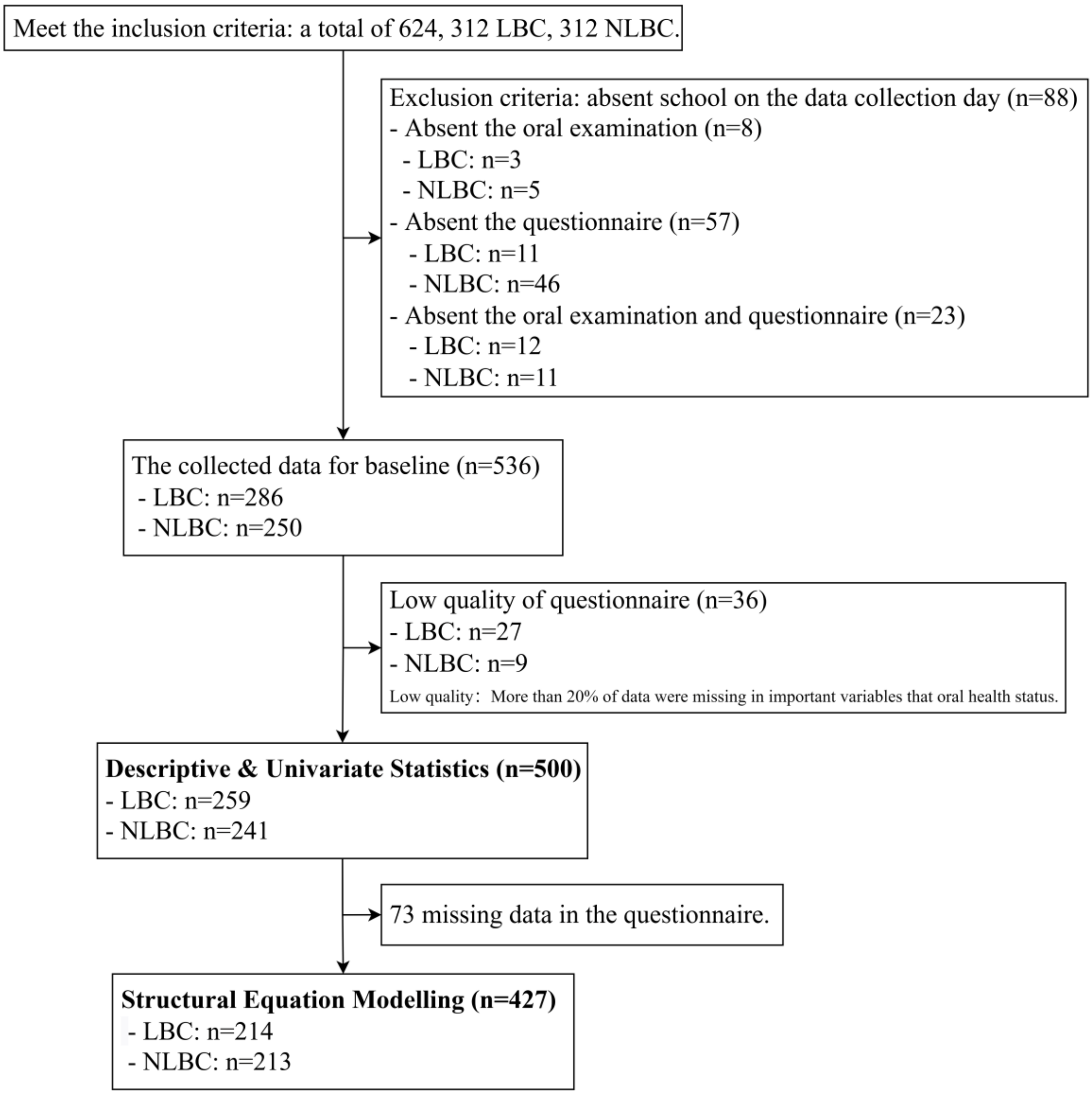

Figure S3. A flow diagram of participants

Table S2. CFA results on latent variables

Factors	Factor loading	Internal consistency reliability	Convergent validity
		Cronbach's alpha	AVE
Acceptable level	> 0.40	> 0.40	> 0.50
Caries in permanent teeth		0.74	0.65
DMFT	0.73		
Presence of permanent teeth caries	1.16		
Parental migration		0.86	0.75
Parental presence	0.90		
Parental migration duration	0.84		
SES		0.43	0.51
Family belongings	0.86		
Education of caregivers	0.35		
Family economic status	0.30		
Snacking consumption		0.64	0.55
Dessert	0.92		
Sugared drink	0.51		

Notes: AVE: Average variance extracted.
